# Latin American undergraduate medical journals

**DOI:** 10.3402/meo.v19.25901

**Published:** 2014-10-21

**Authors:** Patricio Alfaro-Toloza, Romina Olmos-de-Aguilera, Alfonso J. Rodríguez-Morales

**Affiliations:** 1 Asociación Chilena de Seguridad, Chillán, Chile; 2 Faculty of Medicine, Universidad Católica de la Santísima Concepción, Concepción, Chile; 3 Faculty of Health Sciences, Universidad Tecnológica de Pereira, Pereira, Colombia

According to some reports (1), the number of physicians involved in research in developing countries has decreased in recent years. This trend beckons stimulating the interest in medical research early in students’ medical training, including those skills needed to publish and exercise ethical scientific conduct (2, 3).

In Latin America, the last two decades has seen the emergence of student medical journals aimed at developing medical research interest among undergraduates and increasing research and scientific publication (3, 4). Many of these journals arose, in part, from the foundation of the Latin American Federation of Medical Students Scientific Societies (FELSOCEM) which, in 1986, was created to promote and stimulate undergraduate medical research (4).

These peer-reviewed outlets publish original articles, review articles, case reports, letters to the editor, and editorials – and differ from other professional journals only in that they are directed by medical students (4). The origin of these student scientific journals is varied. Some are published only by students from the same university [e.g., *Medico Cientifica* (Panama) or *Ciencia Medica* (Bolivia)], whereas others are national [*ANACEM* (Chile)], or even broader in scope [*CIMEL* (Latin America)].

In Latin America, some student journals have been able to meet quality standards as specified by several respected academic indexes, such as LILACS and SciELO, although they have not yet risen to the levels of internationally acclaimed indexes like ISI or Scopus. Despite this, many of these journals are routinely accessed and cited not only by medical students but also by other Latin American physicians whose searches yield relevant results. Indeed, work appearing in these outlets is sometimes cited in the more prestigious professional journals.

Arguably, one commonly used indicator of quality is a journal's impact factor, which is based on the number of citations. For most Latin American medical student journals, which are not indexed in ISI, this involves the use of an alternative index. The ‘H’ index is based on the distribution of citations received by a given publication. It grows as citations accumulate, and even a low number, for medical students, would be a significant achievement. A scientist, an institution, or a journal has index h if h of his/her Np (number published) papers have at least h citations each, and the other (Np – h) papers have no more than h citations each (5). So, an H index of 10 means that 10 of them have been cited at least 10 or more times by others, regardless of how many papers have been published.

From previously published articles (4), we identified 20 Latin American undergraduate medical journals with exclusively medical student editorial boards. Of these, we examined only those with an H index ≥2. *CIMEL*, the journal of FELSOCEM, had more than 400 citations and an H index of 11 (Table 1). Besides this journal, several others have an H index of four.

**Table 1:** Latin American undergraduate medical journals

Journals	Country	Citations	H index	Founded year
*CIMEL*	Peru	476	11	1995
*Médicas UIS*	Colombia	71	4	1987
*Acta Científica Estudiantil*	Venezuela	66	4	2003
*Revista 16 de Abril*	Cuba	39	4	1961
*Revista ANACEM*	Chile	35	3	2007
*Revista Científica Ciencia Médica*	Bolivia	19	2	1996
*Revista Médico Científica*	Panama	16	2	1980
*Revista SCEMUSS*	Chile	4	2	2005

Annually, there is a meeting of these junior journal editors at the Latin American Scientific Congress of Medical Students. Last year, in Honduras, a consensus was reached on the need to: 1) increase quality/standards toward achieving Scopus indexing; and 2) focus on the ethical aspects of undergraduate scientific publishing (3, 6).

Although modest, these results are encouraging and represent the first bibliometric analysis of Latin American medical student journals. There is still a long road to reach indexation in databases such as Scopus and Web of Science, but quality and performance continue to improve – making them good incubators for future medical editors and, perhaps ultimately, medical researchers.

*Patricio Alfaro-Toloza* Asociación Chilena de Seguridad Chillán, Chile Email: palfarotoloza@gmail.com*Romina Olmos-de-Aguilera* Faculty of Medicine Universidad Católica de la Santísima Concepción Concepción, Chile*Alfonso J. Rodríguez-Morales* Faculty of Health Sciences Universidad Tecnológica de Pereira Pereira, Colombia
